# Immunoglobulin G4 (IgG4)-Related Disease With a Biphasic Clinical Course of Spontaneous Remission and Relapse: A Case Report of Diagnostic Reasoning

**DOI:** 10.7759/cureus.115064

**Published:** 2026-08-24

**Authors:** Hideo Kidogawa, Takashi Okimoto, Yurika Fukudome, Junya Noguchi, Takatomo Yamayoshi

**Affiliations:** 1 Department of Surgery, Kitakyushu City Yahata Hospital, Fukuoka, JPN

**Keywords:** autoimmune pancreatitis, covid-19 mrna vaccine, drug-induced liver injury, enzyme dissociation, igg4-related sclerosing cholangitis

## Abstract

Immunoglobulin G4-related disease (IgG4-RD) can affect multiple organs, but reports of a biphasic clinical course characterized by metachronous multiorgan involvement, where distinct organ lesions emerge sequentially separated by a prolonged latent period, are uncommon. We report the case of a 46-year-old Japanese man. On Day 1, he developed autoimmune pancreatitis (AIP), which achieved clinical and radiological spontaneous remission without any immunosuppressive treatment. After an asymptomatic latent period of approximately 2.5 years (Month 32), he developed a metachronous relapse presenting as IgG4-related sclerosing cholangitis (IgG4-SC). Initial blood tests at our department during the relapse revealed a distinct enzyme dissociation: a markedly elevated gamma-glutamyl transferase (GGT) level (1,569 U/L) juxtaposed with a disproportionately lower alkaline phosphatase elevation (261 U/L). Based on characteristic magnetic resonance cholangiopancreatography findings, markedly elevated serum total IgG (2,155 mg/dL) and IgG4 (573 mg/dL), and a documented history of AIP (where initial endoscopic ultrasound-guided fine-needle aspiration had excluded pancreatic malignancy), a clinical diagnosis of IgG4-SC was established. Treatment with oral prednisolone (PSL; 40 mg/day) was initiated, resulting in rapid biochemical, morphological, and clinical improvement. During the steroid tapering phase, the patient developed trimethoprim-sulfamethoxazole-induced liver injury. This was accurately differentiated from disease relapse and successfully managed by promptly discontinuing the offending drug. The patient is currently maintaining clinical remission on a maintenance dose of PSL (5 mg/day). This case highlights an uncommon biphasic and metachronous course of IgG4-RD. It underscores the importance of precise diagnostic reasoning by integrating the patient's previous clinical history, recognizing enzyme dissociation patterns, and astutely differentiating drug-induced liver injury to prevent unnecessary steroid escalation during long-term management.

## Introduction

Immunoglobulin G4-related disease (IgG4-RD) is a systemic fibroinflammatory condition characterized by elevated serum IgG4 concentrations and dense lymphoplasmacytic infiltrates rich in IgG4-positive plasma cells in the affected organs [1]. IgG4-related sclerosing cholangitis (IgG4-SC) represents the biliary manifestation of this disease and frequently co-occurs synchronously with autoimmune pancreatitis (AIP) in 80%-90% of cases [2]. Although spontaneous clinical and radiological remission in untreated Type 1 AIP has been documented in approximately 40% of patients in historical cohorts [3], the subsequent metachronous development of extrapancreatic disease-specifically presenting as isolated IgG4-SC after a prolonged, untreated asymptomatic latent interval of 2.5 years-represents an exceptionally rare clinical course.

Herein, we report an uncommon case of a patient who initially developed AIP that spontaneously remitted without immunosuppressive treatment, followed by a metachronous relapse presenting as IgG4-SC after an asymptomatic latent interval of approximately 2.5 years. Through the clinical trajectory of this case, we discuss the long-term natural history of IgG4-RD, multimodal diagnostic reasoning (incorporating distinct enzyme dissociation patterns), and the crucial differentiation between intercurrent drug-induced liver injury (DILI) and disease relapse during steroid maintenance therapy.

## Case presentation

A 46-year-old Japanese man presented to our department on Day 1 complaining of epigastric pain that had started six days prior. He had no significant past medical history other than having received a two-dose primary series of an unspecified COVID-19 messenger RNA (mRNA) vaccine (approximately 19 and 18 months prior to Day 1). He had not received any subsequent booster doses, citing no specific reason. He had no history of habitual alcohol consumption (occasional drinker only) and was not taking any regular medications, over-the-counter drugs, or herbal supplements prior to presentation.

Initial blood tests on Day 1 (Table 1) revealed elevated pancreatic enzymes: amylase 173 U/L and lipase 485 U/L. Conversely, hepatobiliary enzymes were within normal limits. The serum IgG4 level was elevated at 224.0 mg/dL (1.85 times the upper limit of normal (ULN; 121 mg/dL)). Serological screening for viral hepatitis (hepatitis B surface antigen and anti-hepatitis C virus antibody) was negative.

**Table 1:** Longitudinal laboratory data during the first phase (autoimmune pancreatitis) showing spontaneous remission ALP, alkaline phosphatase; ALT, alanine aminotransferase; AST, aspartate aminotransferase; CRP, C-reactive protein; EUS-FNA, endoscopic ultrasound-guided fine-needle aspiration; GGT, gamma-glutamyl transferase; H, high (above reference range); IgG4, immunoglobulin G4; WBC, white blood cell count

Laboratory parameter	Reference range	Day 1 (initial presentation)	EUS-FNA admission (Day 21)	Remission confirmed (Day 90)
Total protein (g/dL)	6.6-8.1	7.6	7.8	7.7
Albumin (g/dL)	4.1-5.1	4.6	4.6	4.6
Total bilirubin (mg/dL)	0.4-1.5	0.5	0.5	0.6
AST (U/L)	13-30	16	14	19
ALT (U/L)	10-42	14	17	19
ALP (U/L)	38-113	67	76	58
GGT (U/L)	13-64	30	26	33
Amylase (U/L)	44-132	173 H	138 H	85
Lipase (U/L)	13-55	485 H	-	82 H
WBC (×10^3^/μL)	3.3-8.6	7.5	7.8	8.5
CRP (mg/dL)	< 0.14	0.21 H	0.06	0.02
IgG4 (mg/dL)	11.0-121.0	224.0 H	-	-

**Figure 1:**
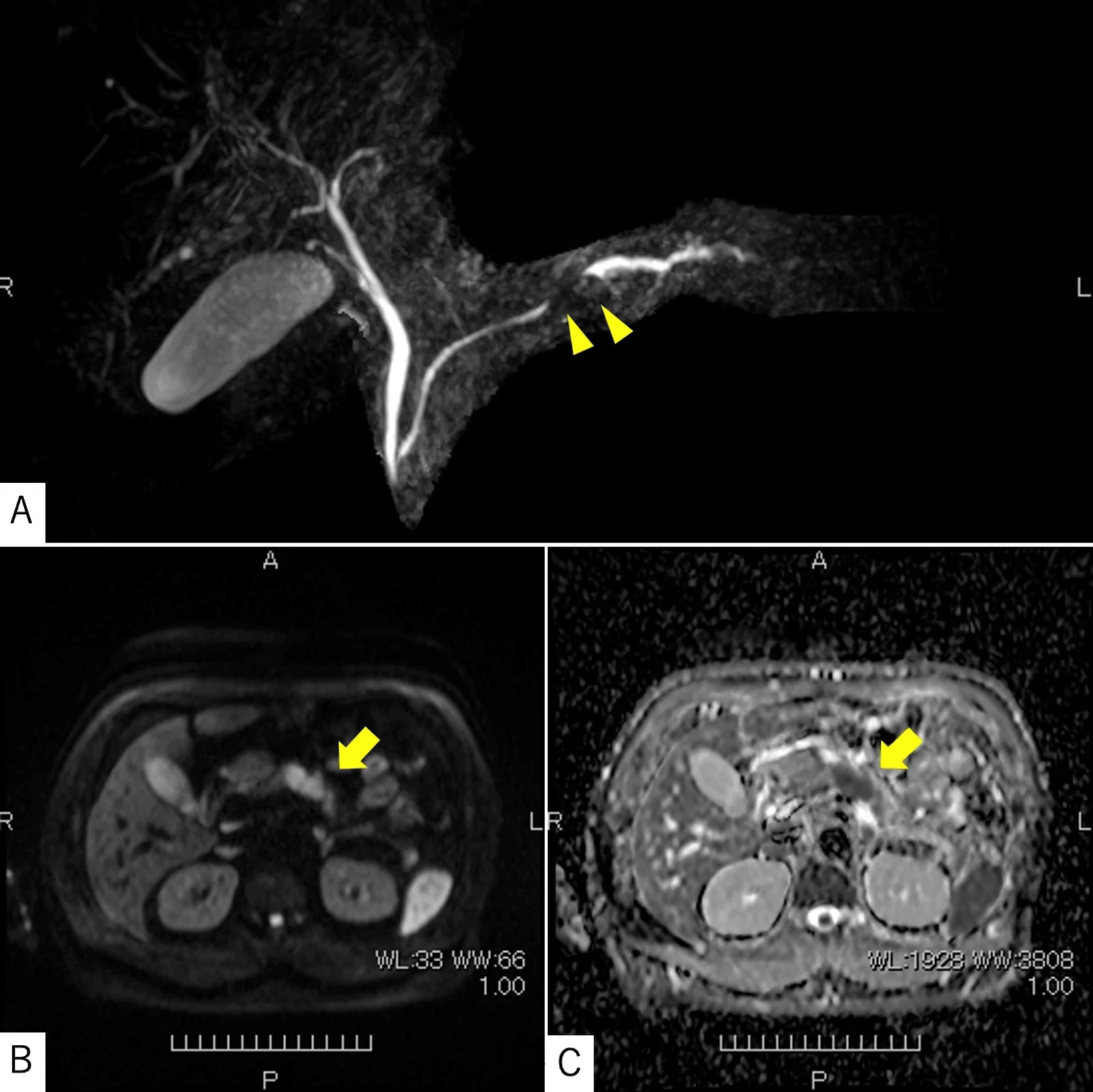
Abdominal magnetic resonance imaging findings at initial presentation (Day 1) (A) Magnetic resonance cholangiopancreatography demonstrating mild dilation of the distal main pancreatic duct (arrowheads). (B) Diffusion-weighted imaging showing a 4 × 2 cm mass in the pancreatic body with high signal intensity (arrow). (C) An apparent diffusion coefficient map revealing a corresponding signal reduction in the same lesion (arrow)

Magnetic resonance imaging (MRI) of the abdomen revealed a 4 × 2 cm mass in the pancreatic body, accompanied by mild dilation of the distal main pancreatic duct. The mass exhibited high signal intensity on diffusion-weighted imaging (DWI) and a corresponding reduction in the apparent diffusion coefficient (Figure 1). Due to the suspicion of pancreatic ductal adenocarcinoma based on these findings, endoscopic ultrasound-guided fine-needle aspiration (EUS-FNA) was performed primarily to rule out malignancy, yielding Class II cytology (benign inflammatory changes with mature lymphocytes and plasma cells, negative for malignancy). Due to the limited tissue architecture inherent to aspiration cytology, specific IgG4 immunostaining and histological features (storiform fibrosis, obliterative phlebitis) could not be formally evaluated. Based on the International Consensus Diagnostic Criteria (fulfilling Level 2 parenchymal imaging and Level 2 serology) [4] and the Japanese 2018 clinical diagnostic criteria [5], a clinical diagnosis of AIP (probable Type 1 AIP) was established. The patient was managed conservatively without immunosuppressive therapy. By Month 3, his clinical symptoms and repeat abdominal imaging findings had resolved, and his pancreatic enzyme levels had nearly normalized (lipase mildly elevated at 82 U/L), achieving clinical and radiological spontaneous remission without medical intervention (Table 1). Regular follow-up was temporarily concluded.

However, approximately 2.5 years after the remission of AIP, liver dysfunction was incidentally detected during a routine health check-up at Month 32. An abdominal ultrasound performed at the primary care clinic revealed a patchy fatty liver, multiple dilations of the peripheral intrahepatic bile ducts, and suspected mild wall thickening at the hepatic hilum without obvious hilar dilation. Based on these findings and his history of AIP, the referring physician suspected an autoimmune-mediated cholangitis and directed the patient to our department.

**Figure 2:**
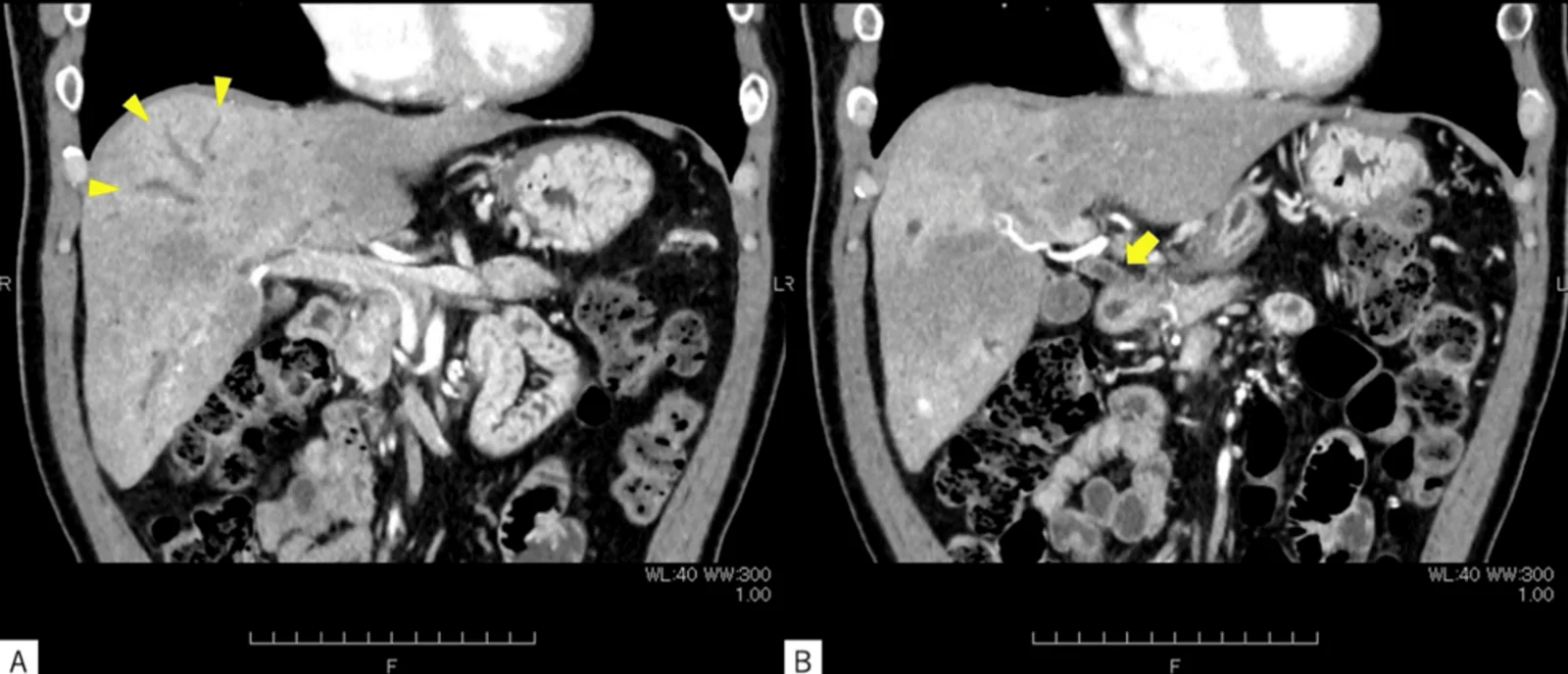
Contrast-enhanced CT findings at the time of relapse (Month 32) (A) Coronal arterial-phase image demonstrating multiple dilations of the peripheral intrahepatic bile ducts (arrowheads). (B) A different coronal section in the same phase shows diffuse wall thickening of the common bile duct (arrow) CT: computed tomography

**Figure 3:**
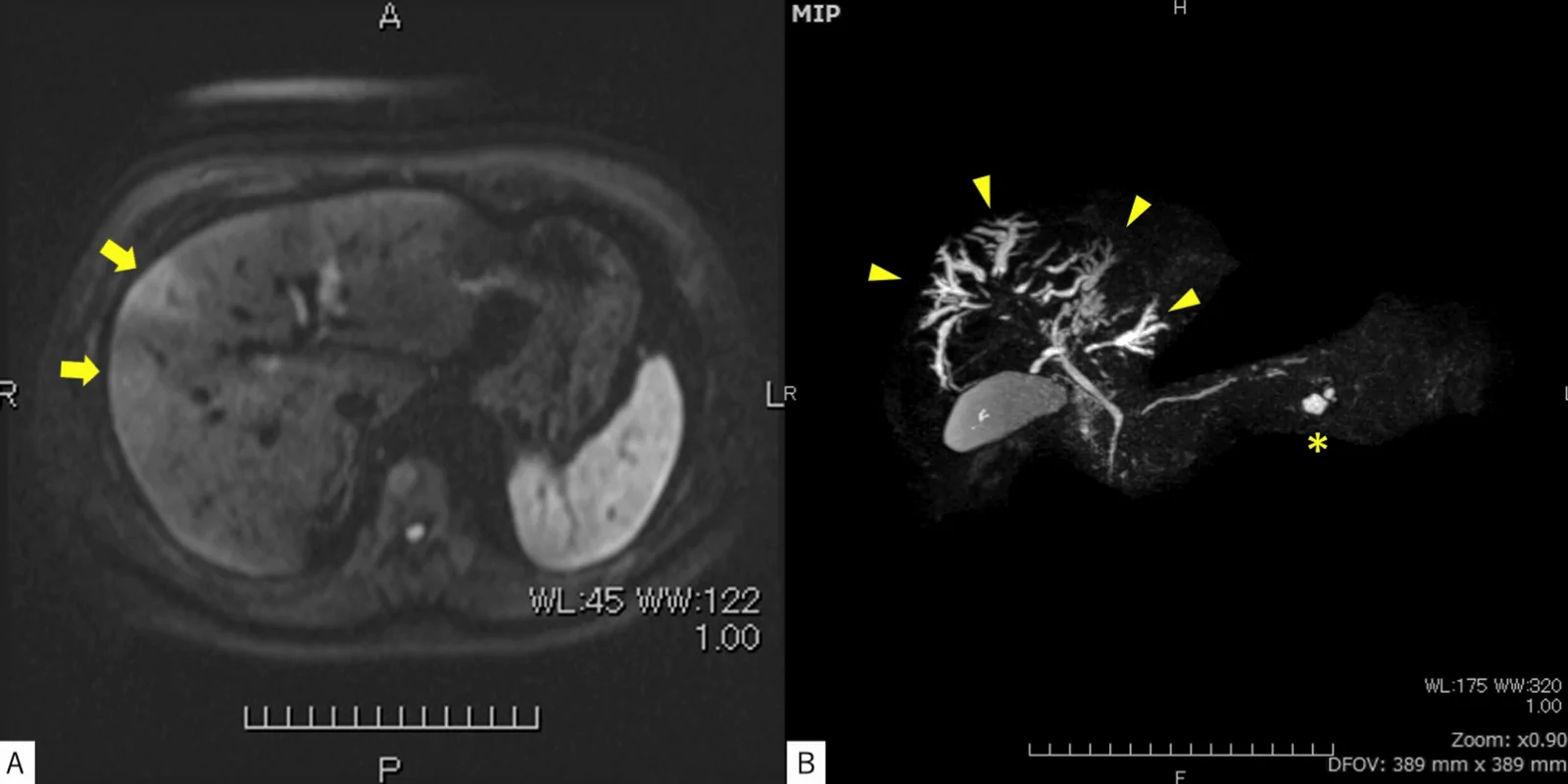
Magnetic resonance imaging and MRCP findings at the time of relapse (Month 32) (A) Axial diffusion-weighted imaging revealing high signal intensity along the peripheral intrahepatic bile ducts in the right hepatic lobe (arrows), indicating active inflammation. (B) Maximum intensity projection MRCP image demonstrating multiple smooth strictures in the intrahepatic and extrahepatic bile ducts, accompanied by upstream dilation (arrowheads). A cystic lesion is also clearly noted in the pancreatic tail (asterisk) MRCP: magnetic resonance cholangiopancreatography

Upon revisiting our department in late Month 32, his laboratory evaluation revealed a distinct enzyme dissociation between GGT and alkaline phosphatase (ALP): a markedly elevated GGT level (1,569 U/L) juxtaposed with a disproportionately low ALP level (261 U/L). Transaminases were also elevated (aspartate aminotransferase (AST) 110 U/L, ALT 172 U/L). Additionally, his serum total IgG level was elevated at 2,155 mg/dL, and his serum IgG4 level was markedly elevated at 573.0 mg/dL (> 4× ULN). Contrast-enhanced computed tomography (CT) performed on the same day revealed diffuse, smooth thickening of the bile duct wall with arterial-phase enhancement (Figure 2), while MRI and magnetic resonance cholangiopancreatography (MRCP) performed a few days later demonstrated restricted diffusion on DWI reflecting active inflammation, along with multiple smooth strictures in the intrahepatic and extrahepatic bile ducts with upstream dilation (Figure 3). Additionally, a small cystic lesion was incidentally noted in the pancreatic tail, presumed to be a benign inflammatory retention cyst secondary to localized ductal inflammation.

According to the "Clinical Diagnostic Criteria for IgG4-Related Sclerosing Cholangitis 2020" (IgG4-SC 2020) [6], the patient fulfilled Criterion 1 (characteristic biliary imaging showing diffuse smooth strictures and wall thickening without an invasive mass), Criterion 2 (marked serum IgG4 elevation >4× ULN), and Criterion 3 (documented prior history of AIP). Based on these validated consensus criteria and the lack of malignant imaging features, a definitive clinical diagnosis of IgG4-SC was established without repeat invasive transluminal biopsy. Treatment with oral prednisolone (PSL) 40 mg/day was initiated following the MRCP. Prophylactic trimethoprim-sulfamethoxazole (TMP-SMX) was concurrently administered to prevent opportunistic infections.

Clinical course and management

Following initiation of PSL therapy, both serum IgG and GGT levels declined rapidly. However, during the steroid tapering phase (PSL 10 mg/day) at Week 16 of steroid therapy, an acute elevation of transaminases (AST 61 U/L, ALT 148 U/L) was observed, while disease activity markers (IgG 1,031 mg/dL, GGT 633 U/L) remained stable. An R ratio of 3.02 indicated a mixed-type liver injury. Given the stable IgG levels and the absence of cholestatic resurgence, TMP-SMX-induced DILI was suspected rather than a relapse of IgG4-SC, and the drug was discontinued. Transaminase levels promptly decreased within two weeks (AST 35 U/L, ALT 68 U/L).

At the subsequent follow-up at Week 26 of steroid therapy, the transaminases had completely normalized, confirming the diagnosis of DILI and the absence of primary disease relapse. Although serum GGT remained moderately elevated at 439 U/L, this represented a marked reduction from the peak (1,569 U/L). Coupled with normalized transaminases, normal IgG levels, and symptom resolution, the patient achieved and maintained clinical remission. The detailed longitudinal laboratory data from the initiation of steroid therapy are summarized in Table 2.

**Table 2:** Longitudinal laboratory findings following the initiation of systemic steroid therapy The transaminase elevation observed at Week 16 of steroid therapy was attributed to TMP-SMX-induced liver injury. Levels rapidly improved upon discontinuation of the drug and achieved complete normalization by Week 26 of steroid therapy ALP, alkaline phosphatase; ALT, alanine aminotransferase; AST, aspartate aminotransferase; CRP, C-reactive protein; DILI, drug-induced liver injury; GGT, gamma-glutamyl transferase; H, high (above reference range); IgG, immunoglobulin G; IgG4, immunoglobulin G4; L, low (below reference range); TMP-SMX, trimethoprim-sulfamethoxazole; WBC, white blood cell count

Laboratory parameter	Reference range	Baseline (Month 32)	Week 4	Week 10	Week 16 (DILI onset)	Week 18 (DILI recovery)	Week 26 (clinical remission)
Prednisolone dose (mg/day)	-	0	40	20	10	10	5
IgG (mg/dL)	861-1,747	2,155 H	1,024	-	1,031	1,051	-
IgG4 (mg/dL)	11.0-121.0	573.0 H	-	-	-	-	-
AST (U/L)	13-30	110 H	32 H	32 H	61 H	35 H	21
ALT (U/L)	10-42	172 H	103 H	87 H	148 H	68 H	39
ALP (U/L)	38-113	261 H	110	118 H	132 H	122 H	104
GGT (U/L)	13-64	1,569 H	876 H	783 H	633 H	592 H	439 H
Amylase (U/L)	44-132	41 L	48	58	44	57	50
WBC (×10^3^/μL)	3.3-8.6	7	13.0 H	11.6 H	9.2 H	9.1 H	8.6
CRP (mg/dL)	<0.14	-	<0.01	-	0.24 H	0.04	-

**Figure 4:**
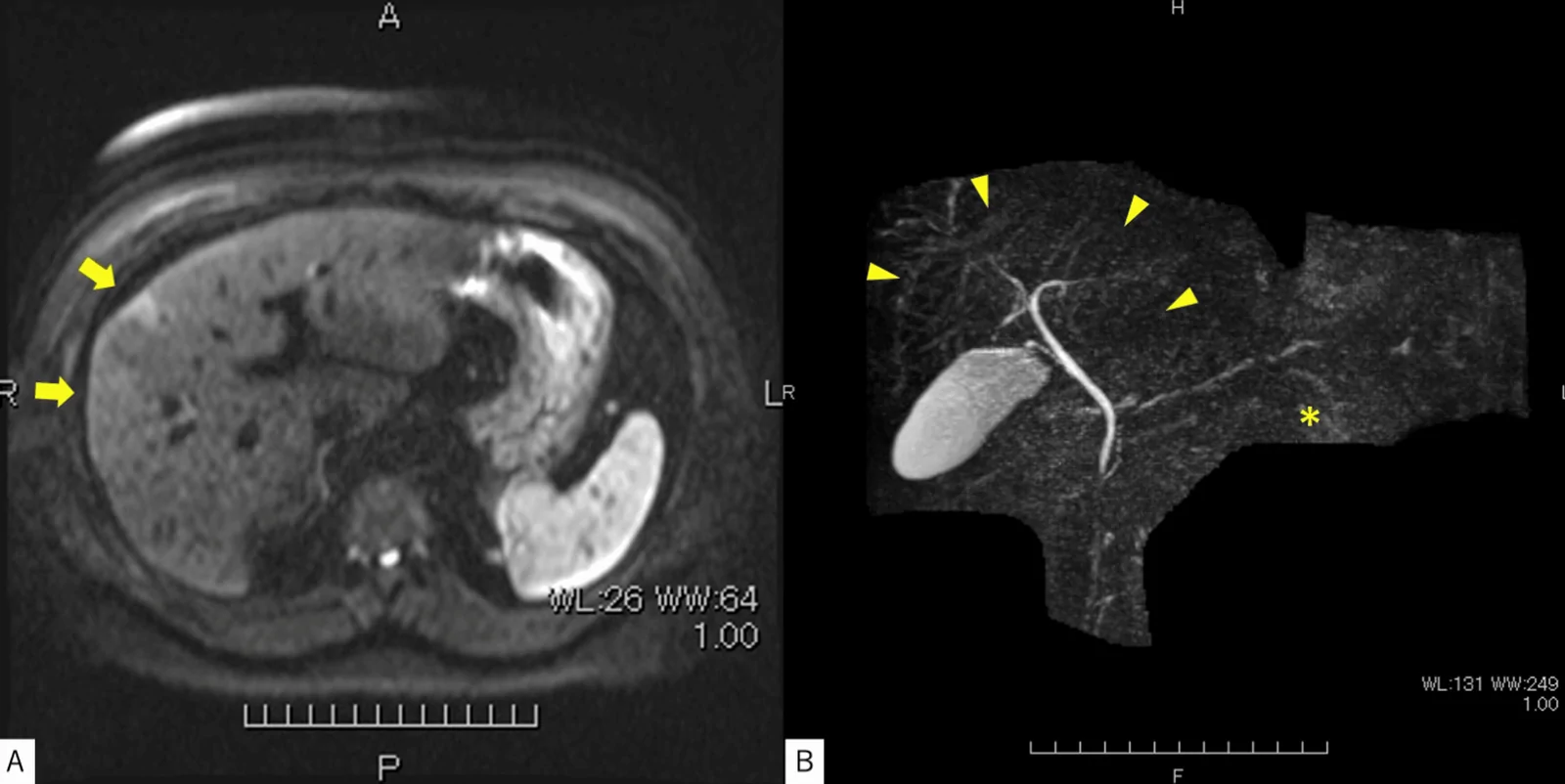
Follow-up magnetic resonance imaging and MRCP findings after steroid therapy (at Week 30 of steroid therapy) (A) Axial diffusion-weighted imaging showing minor residual hyperintensity in the hepatic parenchyma (arrows). (B) Follow-up MRCP demonstrating marked improvement of intrahepatic bile duct dilations and strictures (arrowheads), as well as the complete disappearance of the previously noted cystic lesion in the pancreatic tail (asterisk) MRCP: magnetic resonance cholangiopancreatography

Furthermore, follow-up MRI and MRCP performed at Week 30 of steroid therapy confirmed marked morphological improvement, including the complete resolution of biliary strictures and the disappearance of the pancreatic tail cyst, corroborating systemic steroid-responsive remission (Figure 4). The patient currently maintains clinical and morphological remission on a maintenance PSL dose of 5 mg/day without signs of relapse or steroid withdrawal symptoms.

## Discussion

This case provides valuable insights into the natural history, diagnostic reasoning, and clinical management of IgG4-RD. The clinical trajectory encompasses several notable aspects: the spontaneous remission of AIP followed by metachronous multiorgan involvement presenting as IgG4-SC, a distinct enzyme dissociation pattern, the differentiation of intercurrent adverse drug reactions during therapy, and the long-term management of chronic autoimmune disease.

Recently, emerging literature has documented cases of IgG4-RD (such as AIP and IgG4-related hepatopathy) developing after COVID-19 mRNA vaccination [7,8]. While most previously reported cases exhibited an acute or subacute onset within days to weeks postvaccination, our patient presented with an unusually extended clinical timeline: approximately 1.5 years elapsed between the primary two-dose vaccination series and the initial AIP onset (Day 1), followed by an additional 2.5-year latent period prior to the metachronous IgG4-SC relapse (Month 32). Given this prolonged latency and the absence of booster doses, establishing a direct, acute causal relationship is challenging, and coincidental occurrence cannot be ruled out. Nevertheless, repetitive antigen exposure and mRNA platforms have been demonstrated to induce unique immune modifications, including persistent antibody class-switching toward IgG4 [9]. In the pathophysiology of IgG4-RD, progressive immune dysregulation and epitope spreading often evolve insidiously over extended periods. Therefore, while a direct causal link cannot be definitively proven from a single case, the possibility that prior mRNA vaccination exerted an "immunological priming" effect-contributing to a subtle, long-term breakdown of immune tolerance in a genetically or immunologically susceptible individual-remains an intriguing hypothesis that cannot be entirely excluded. The accumulation of similar longitudinal cases and mechanistic studies is warranted to elucidate the potential long-term immunological sequelae of mRNA vaccines.

A notable diagnostic clue during the relapse was the distinct enzyme dissociation: a marked elevation of GGT (1,569 U/L) juxtaposed with a disproportionately lower ALP level (261 U/L). In classical cholestatic conditions such as primary sclerosing cholangitis or malignant biliary obstruction, both GGT and ALP typically rise in tandem. However, as Tanaka [10] observed, an isolated or predominant elevation of GGT relative to ALP can be encountered in active IgG4-SC. While the exact mechanism remains an exploratory hypothesis rather than an established diagnostic hallmark, this enzyme dissociation may reflect acute periductal lymphoplasmacytic inflammation and microenvironmental epithelial stress prior to the development of widespread mechanical obstruction and established chronic cholestasis. In our patient, recognizing this enzyme dissociation pattern prompted detailed biliary imaging and prevented misattributing the liver dysfunction solely to alcoholic liver injury or non-specific steatohepatitis. Although baseline hepatic steatosis identified on ultrasonography may have served as a minor contributing factor, the massive spike in GGT (1,569 U/L) predominantly reflected active IgG4-SC.

A critical challenge in evaluating IgG4-SC is the rigorous exclusion of pancreatobiliary malignancies, particularly cholangiocarcinoma [6,11]. In this patient, although direct transluminal biliary biopsy was not performed during the second episode, a definitive diagnosis of IgG4-SC was established according to the validated "Clinical Diagnostic Criteria for IgG4-Related Sclerosing Cholangitis 2020" (IgG4-SC 2020) [6]. Malignancy was comprehensively ruled out based on characteristic imaging features (diffuse, smooth concentric wall thickening without abrupt eccentric strictures or invasive mass on CT and MRCP), a markedly elevated serum IgG4 level (>4× the ULN, which confers high specificity against cholangiocarcinoma), a documented prior history of AIP (in which baseline pancreatic malignancy was excluded by EUS-FNA), and rapid radiological resolution following steroid initiation. Additionally, the complete resolution of the incidental pancreatic tail cyst following corticosteroid therapy supports the interpretation that it represented a benign, IgG4-related inflammatory retention cyst secondary to localized ductal inflammation.

Finally, distinguishing between DILI and disease relapse during steroid tapering represents a vital aspect of long-term management. While corticosteroids are highly effective in inducing remission in IgG4-RD, disease flare-ups frequently occur during dose reduction [12]. When an acute elevation of transaminases occurred at a PSL dose of 10 mg/day (Week 16), reflexively assuming a disease relapse and escalating immunosuppressive therapy could have exposed the patient to unnecessary drug toxicity and increased the risk of opportunistic infections. By carefully analyzing the liver enzyme kinetics, specifically, an isolated transaminase elevation with an R ratio of 3.02 (indicating mixed-type liver injury) in the setting of stable disease activity markers (normal serum IgG levels and declining GGT), we deduced that the hepatic insult was TMP-SMX-induced DILI rather than active IgG4-SC recurrence. Prompt discontinuation of the offending antibiotic led to rapid biochemical recovery within two weeks and subsequent complete normalization of transaminases, enabling successful continuation of steroid tapering to a low maintenance dose. Differentiating intercurrent adverse drug reactions from true autoimmune relapse through multimodal clinical reasoning is essential for optimizing patient safety during long-term maintenance therapy [13].

Limitations

This study has several limitations. First, causality between the COVID-19 mRNA vaccination and the clinical onset of IgG4-RD cannot be established. Given the prolonged latency, any immunological association remains purely speculative based on a single case report (n = 1). Second, the precise pathophysiological mechanism underlying the marked GGT/ALP enzyme dissociation observed during the IgG4-SC episode remains unproven and should be regarded as an exploratory observation. Third, serum IgG4 levels were not measured during the initial spontaneous remission phase at Month 3; thus, confirmation of initial remission relied on clinical symptom resolution, pancreatic enzyme normalization, and imaging findings without serological corroboration. Fourth, histological confirmation of IgG4-SC was unavailable, as the diagnosis was established clinically using validated noninvasive consensus criteria. Fifth, histological reassessment and direct transluminal biliary biopsy were not performed during the relapse at Month 32. Although the negative EUS-FNA at Day 1 excluded malignancy in the initial pancreatic mass, it did not histologically rule out a newly developed biliary malignancy (such as cholangiocarcinoma) at relapse. Instead, the exclusion of malignancy and the diagnosis of IgG4-SC were established based on comprehensive clinical, biochemical, and radiological criteria, specifically, characteristic smooth biliary strictures on MRCP/CT, serum IgG4 >4× ULN, documented history of AIP, and prompt therapeutic response to corticosteroids, in accordance with validated clinical guidelines. Sixth, hepatic steatosis identified on ultrasonography represents a potential confounding factor when interpreting serum GGT levels, although its contribution to the acute, severe enzyme surge was considered secondary.

## Conclusions

In conclusion, this case illustrates an uncommon biphasic and metachronous clinical course of IgG4-RD, characterized by the initial spontaneous remission of AIP followed by the delayed emergence of IgG4-SC after a 2.5-year latent period. This report delivers three practical lessons for clinical practice. First, clinicians should maintain long-term vigilance for delayed, metachronous multiorgan involvement even in patients who have achieved apparent spontaneous resolution without immunosuppressive therapy. Second, establishing an accurate diagnosis of IgG4-SC requires a comprehensive diagnostic reasoning process that integrates high-resolution biliary imaging, marked serum IgG4 elevation, and prior clinical history, while carefully interpreting suggestive clinical clues such as GGT/ALP enzyme dissociation. Third, and most importantly, when acute liver dysfunction arises during steroid tapering, clinicians must avoid reflexively attributing the enzyme elevation to disease relapse and unnecessarily escalating immunosuppression. Instead, intercurrent DILI, such as that caused by TMP-SMX used for Pneumocystis pneumonia prophylaxis-should be actively considered, and a meticulous evaluation integrating liver enzyme kinetics and stable serological disease activity markers (IgG) is essential to guide prompt drug withdrawal and protect patient safety.
